# The reciprocal relationships between meaning in life and smartphone addiction among Chinese college students: evidence from a three-wave cross-lagged panel model

**DOI:** 10.3389/fpubh.2023.1202741

**Published:** 2023-07-13

**Authors:** Hao Zhao, Tianjiao Song, Shameem Rafik-Galea, Jihe Dong, Mimi Fitriana, Yanhong Ji, Jianling Zhang

**Affiliations:** ^1^School of Education, Shandong Women's University, Jinan, China; ^2^Faculty of Education, Languages, Psychology and Music, SEGI University, Kuala Lumpur, Malaysia; ^3^Faculty of Arts and Science, International University of Malaya-Wales, Kuala Lumpur, Malaysia

**Keywords:** Chinese college students, reciprocal relationships, meaning in life, smartphone addiction, cross-lagged

## Abstract

**Background:**

Previous cross-sectional studies have shown that meaning in life (MIL) is closely associated with college students’ smartphone addiction (SA), but the causal relationship between MIL and college students’ SA is uncertain. Therefore, conducting a longitudinal study to explore their relationship is very necessary. Furthermore, some studies have implied possible gender differences in the relationship between MIL and SA and the relationship between SA and MIL. Therefore, it is necessary to further examine whether there are gender differences in the above relationships.

**Methods:**

The present study constructed a three-wave cross-lag panel model to explore the relationships between MIL and college students’ SA. Three waves of data were collected from 705 college students (male: 338; female: 367) in China for three consecutive years, and the interval of data collection was 1 year. These college students completed the same online questionnaire regarding MIL and SA.

**Results:**

(1) The MIL of male college students was significantly stronger than that of female college students at time 1, time 2, and time 3, (2) Female college students’ SA at time 1, time 2, and time 3 was more serious than that of male college students, (3) There were reciprocal relationships between MIL and college students’ SA, (4) The influence of MIL on female college students’ SA was significantly stronger than that of male college students, and (5) The influence of SA on female college students’ MIL was significantly stronger than that of male college students.

**Conclusion:**

This study showed reciprocal relationships between MIL and SA among male college students and female college students. The findings further deepen our understanding of the relationship between MIL and SA and provide a gender perspective for preventing or intervening with college students’ SA.

## 1. Introduction

According to the 50th Statistical Report on Internet Development in China, as of June 2022, there were 1.047 billion smartphone users in China, making up of 99.6% of all Internet users (1). Because of its portability, interaction, and immediacy, smartphones have become a daily necessity for people in modern society. However, frequent smartphone use can quickly result in smartphone addiction (2). Smartphone addiction is similar to Internet addiction and game addiction, and it is defined by Sunday et al. (3) as “a condition where the use of smartphone has fulfilled a deep need (dependency, habitual, and addictive behavior) to the extent that the individual has difficulty conducting basic activities of daily life without the concurrent use of a smartphone, and as such caused neglect of other aspects of one’s life.” Smartphone addiction may lead to a series of physiological symptoms (e.g., vision loss, headaches, and decreased sleep quality) and psychological symptoms (e.g., memory loss, emotional depression, and anxiety) (4). A study on smartphone usage among Chinese college students found that more than 20% of college students use their smartphones for more than 7 h a day (5). Numerous studies in different countries and cultures have revealed that college students are a high-risk group of SA (6–13). SA does great harm to college students. It is difficult for college students with SA to concentrate on their studies (14), and their happiness in life is significantly reduced due to the excessive use of smartphones (15–17). In severe cases, SA can cause personality disorder (18, 19) and even suicidal tendencies (20–22). Therefore, it is very essential to pay close attention to college students’ SA and deeply explore the causes of college students’ SA so as to provide theoretical and methodological support for effective intervention or prevention of college students’ SA.

Meaning in life (MIL) refers to “the sense made of, and significance felt regarding, the nature of one’s being and existence” (23). MIL is closely associated with addictive behaviors. Numerous studies have revealed that the higher an individual’s MIL, the less likely he or she is to develop addictive behaviors (24–29). According to meaning therapy theory, meaning in life is the basic element of happiness in life experienced by individuals, and constantly seeking the meaning and purpose of life is the fundamental drive of human existence (30). The lack of meaning in life may cause individuals to fall into a state of emptiness, leading them to lose themselves in real life, which in turn leads to addictive behaviors and then seriously affects the physical and mental health of individuals (30). The meaning therapy theory is supported by some empirical studies among college students. Until now, many studies have indicated that MIL is closely related to college students’ SA. Specifically, studies have revealed that college students’ MIL and their level of SA are negatively associated (28, 29). MIL not only has a direct effect on college students’ SA, but also has an indirect effect on college students’ SA through other variables. For example, some scholars verified that MIL could indirectly affect college students’ SA through self-control (31). In addition, a recent research has shown that MIL can indirectly affect college students’ SA through school adjustment (32). Summarizing the above literature, many scholars have made beneficial investigations on the relationship between MIL and college students’ SA, but these studies only investigated the influence of MIL on college students’ SA, and all the studies were cross-sectional rather than follow-up studies.

From the current studies, although there is no direct study on the influence of SA on college students’ MIL, the resource conservation theory and some related studies suggest that the above influence is likely to exist. According to the resource conservation theory proposed by Hobfoll (33), individuals have limited resources (e.g., time, energy, and attention), and people will strive to maintain, protect, and acquire these resources. However, the loss of these resources is likely to pose a threat to people’s lives, resulting in negative consequences. The above notion has been supported by some empirical studies. These studies have found that SA is significantly related to college students’ interpersonal problems (34, 35) and self-esteem (36, 37). SA can trigger college students’ anxiety (38, 39) and depression (36, 40–42). Furthermore, a longitudinal study revealed that SA could significantly positively affect adolescents’ depression (43). Since interpersonal relationships, self-esteem, anxiety, and depression are all important predictors of college students’ MIL (29, 44–48), based on the resource conservation theory, we speculate that SA is also probably an important influencing factor of MIL. In short, from the previous studies, the causal relationship between MIL and college students’ SA is not clear. Few studies have explored the reciprocal relationships between MIL and college students’ SA.

In addition to exploring the relationship between MIL and college students’ SA, scholars have also conducted some studies on gender differences in college students’ MIL and SA. On the whole, previous studies have revealed that female college students’ meaning in life is significantly lower than that of male college students (49–52), and female college students’ smartphone addiction is more serious than that of male college students (53–59). Males and females are very different when it comes to coping with external pressures. Compared with males, females may perceive threatening events as stress events and often feel in a stressful environment (60–63). There are obvious differences between males and females in coping with external pressure. Males are more focused on problem solving, and may be more inclined to use positive coping styles (e.g., rational coping and detached coping) to cope with pressure, while females may be more inclined to use negative coping styles (e.g., emotional response and avoidance) to cope with pressure rather than focus on problem solving (64, 65). Since negative coping style is a risk factor for the individual’s meaning in life (66, 67), it is understandable why female college students have a lower level of MIL than male college students. Smartphones have the following functional characteristics: (a) collectivity of functions (68, 69); (b) personalization and customization of content (9, 54, 70); (c) accessibility (2, 71); and (d) convenience (2, 71). Some scholars have found that it is precisely because of the above-mentioned functional characteristics of smartphones that female college students use smartphones more frequently in order to make up for the psychological emptiness caused by the low level of MIL and alleviate their negative emotions, thus leading to more serious SA among female college students than male college students (32). However, few studies have explored the moderating effect of gender on the relationship between college students’ MIL and SA.

There may also be gender differences in the relationship between SA and college students’ MIL. The social replacement hypothesis suggests that indulging in social communication through the internet reduces the time for individuals to interact with friends and family, which leads to a small social circle and may lead to depression and loneliness (72). Replacement means that personal use of the Internet may replace offline communication (73, 74). Consistent with the social replacement hypothesis, some studies revealed that individuals may perceive less support from others through Internet communication than offline communication (75, 76). Online social interaction cannot replace offline social interaction: they are not the same psychologically, and addiction to mobile social media may increase the degree of individual social isolation (77). Previous studies have found that female college students will use social media more than male college students to satisfy their social needs (55, 78, 79). Therefore, smartphone addiction may have a more negative impact on female college students’ meaning in life. However, few studies have explored the moderating effect of gender on the relationship between college students’ SA and MIL.

**Figure 1:**
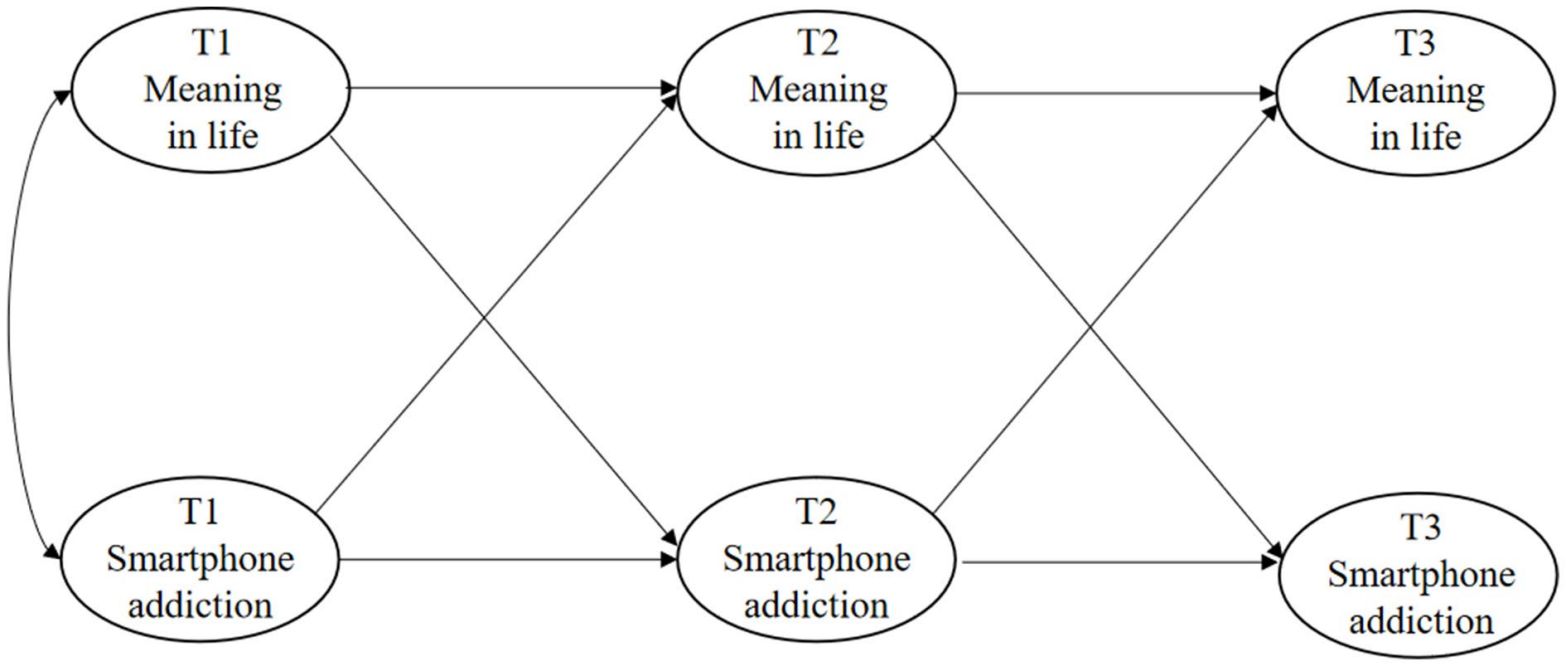
Conceptual model.

In this study, college students are taken as the subjects to conduct a three-wave cross-lag panel model to explore the reciprocal relationships between college students’ MIL and SA (see Figure 1). This study proposed two hypotheses:

*Hypothesis 1*: There are reciprocal relationships between MIL and SA.

*Hypothesis 2*: Gender moderates the reciprocal relationships between MIL and SA.

## 2. Materials and methods

### 2.1. Participants and procedure

Using the convenient cluster sampling method, freshmen from 20 classes at three universities in Shandong Province, China were selected for a 2-year follow-up study in this study, with data collected three times at 1-year intervals. The inclusion criteria are as follows: (a) 18–25 years old, (b) Full-time college students, (c) Smartphone users, and (d) Voluntary participation in the three questionnaire surveys and signing online informed consent form. Exclusion criteria: (a) Suffering from psychiatric disorders and (b) Suffering from other addictive behaviors. Data were collected in early April of 2020 (Time 1), in early April of 2021 (Time 2), and in early April of 2022 (Time 3). The Wen Juan Xing platform was used to distribute the informed consent form and questionnaire to the 20 classes. 802 freshmen participated in the survey at time 1. In the end, 705 students participated in all three surveys, which is equivalent to a total loss of 97 students with a loss rate of 12.09%. All participants signed the online informed consent form. Table 1 shows the demographic profiles of participants.

**Table 1:** The demographic profiles of participants (*n* = 705).

Variable	Levels	*n*	Percent (%)
Gender	Male	338	47.94
	Female	367	52.06
Major	Engineering	188	26.67
	Science	202	28.65
	Liberal arts	315	44.68
Only child/Non-only child	Only child	318	45.11
	Non-only child	387	54.89
Urban/Rural area	Urban area	303	42.98
	Rural area	402	57.02
Father’ educational level	Junior high school or less	303	42.98
	Senior high school	245	34.75
	College or university	126	17.87
	Postgraduate	31	4.40
Mother’ educational level	Junior high school or less	297	42.13
	Senior high school	281	39.86
	College or university	98	13.90
	Postgraduate	29	4.11
Father’ occupational statuses	Peasant or jobless	51	7.23
	Blue collar	313	44.40
	Professional or semiprofessional	341	48.37
Mother’ occupational statuses	Peasant or jobless	69	9.79
	Blue collar	337	47.8
	Professional or semiprofessional	299	42.41
Household income per month (RMB)	Below 3,000	46	6.52
	3,001–6,000	264	37.45
	6,001–9,000	190	26.95
	9,001–12,000	108	15.32
	Above 12,000	97	13.76

### 2.2. Measures

#### 2.2.1. Meaning in life

This study used the Chinese version of Meaning in Life Scale (80) to evaluate the meaning in life of college students. The scale has 9 items in total, which includes two factors: presence of meaning (e.g., “There is no clear purpose in my life”) and searching for meaning (e.g., “I have found a purpose in life that satisfies me”). Each item is rated on a seven-point Likert scale ranging from 1 (strongly disagree) to 7 (strongly agree). In this study, at time 1, time 2, and time 3, the Cronbach’s α of the scale were 0.88, 0.86, and 0.89, respectively. The construct validity of the scale at time 1, time 2, and time 3 was also good: *χ*^2^/df ≤ 2.258, comparative fit index (CFI) ≥ 0.956, Standardized Root Mean Square Residual (SRMR) ≤ 0.041, Tucker-Lewis Index (TLI) ≥ 0.949, root mean square error of approximation (RMSEA) ≤ 0.066.

#### 2.2.2. Smartphone addiction

This study used the Chinese version of Smartphone Addiction Scale – Short Version to assess the college students’ smartphone addiction. The Chinese version scale was translated from the Smartphone Addiction Scale – Short Version (SAS-SV) compiled by Kwon et al. (69) by forward-backward method. The scale has 10 items in total (e.g., “Feeling impatient and fretful when I am not holding my smartphone”). Each item is rated on a six-point Likert scale ranging from 1 (strongly disagree) to 6 (strongly agree). At time 1, time 2, and time 3, the Cronbach’s α of this scale were 0.85, 0.87, and 0.87, respectively. The construct validity of the scale at time 1, time 2, and time 3 was also good: *χ*^2^/df ≤ 2.631, CFI ≥ 0.946, SRMR ≤ 0.034, TLI ≥ 0.938, RMSEA ≤ 0.062.

### 2.3. Data analysis

The present study used SPSS 26.0 and Mplus 7.11 to analyze the data. The data analysis procedure was as follows:

First, conducting a common method bias analysis with SPSS 26.0. Specifically, all items were subjected to Harman’s One-Factor Test (unrotated exploratory factor analysis) (81). If the variance explained by the first component, among all components with eigenvalues larger than 1, is less than 40%, it suggests that there is no common method bias present in this study (81).

Second, SPSS 26.0 was used to conduct descriptive statistics, implement independent samples t-tests to investigate the gender differences in meaning in life (MIL) and smartphone addiction (SA), and examine correlations between MIL and SA.

Third, in order to test whether there are reciprocal relationships between college students’ MIL and SA, the following four competing models were established: Model 1: No cross-lagged effects (T1MIL → T2SA, T2MIL → T3SA, T1SA → T2MIL and T2SA → T3MIL are dropped). Model 2: Smartphone addiction effects (T1SA → T2MIL and T2SA → T3MIL are dropped). Model 3: Meaning in life effects (T1MIL → T2SA and T2MIL → T3SA are dropped); Model 4: Reciprocal effects (all paths are included). Then, using structural equation model to examine the four competing models to determine whether the reciprocal effects model would best fit the data and significantly better than the other three models. Model fit was determined by the following indices: “Comparative Fit Index (*CFI*), Standardized Root Mean Square Residual (*SRMR*), Tucker-Lewis Index (*TLI*), and Root Mean Square Error of Approximation (*RMSEA*). An acceptable model is determined by the following criteria: *CFI* > 0.90, *SRMR* < 0.05*, TLI* > 0.90, and *RMSEA* < 0.08 (82). In addition, the differences of CFI (△CFI > 0.01) and the *χ*^2^ differences between models (*p* < 0.05) were used to determine the best model (83–85).

Fourth, this study used Mplus 7.11 to conduct multiple group (male vs. female) analysis to test gender differences on reciprocal effects. According to the requirements of multiple group analysis, measurement invariance needs to be tested before comparing the differences of structural equation model in different group (85). This is because only when the relationship between observed variables and potential variables is invariant in all groups that need to be compared can the differences of structural equation model be further compared (85). Specifically, measurement invariance was tested by the following four steps: Step 1: Fitting measurement model for each group, respectively. Step 2: Configural invariance. Step 3: Metric invariance (common loadings across gender). Step 4: Scalar invariance (common loadings and intercepts across). Configural invariance is a prerequisite for testing other invariances, and it is usually used as a baseline model for testing (83–85). Further invariance tests are nested models generated by restricting the corresponding parameters on the basis of the invariance at the previous level, and only if the invariance at the previous level is established can the invariance test be continued at a higher level (85). When scalar invariance (strong invariance) is established, measurement invariance is established (83–85). An acceptable model is determined by the following criteria: *CFI* > 0.90, *SRMR* < 0.05*, TLI* > 0.90, and *RMSEA* < 0.08 (82). The differences of CFI (△CFI < 0.01) and the *χ*^2^ differences between models (*p* > 0.05) were used as the criteria of invariant measurements (83–85). Then, using Mplus 7.11 to compare the differences of reciprocal effects among male college students (group 1) and female college students (group 2).

In this study, before testing the cross-lagged panel model, all the variables were standardized. When testing the reciprocal relationships between college students’ MIL and SA, all demographic variables were set as control variable, and when comparing the gender differences of the reciprocal effects, demographic variables except gender were set as control variable. Besides, 95% confidence interval based on the bias-corrected percentile method with 1,000 bootstrap samples was used to examine the reciprocal effects.

## 3. Results

### 3.1. Common method bias

The results of common method bias analysis showed that the eigenvalues of 18 components exceeded 1, and the first component explained 21.36% of the total variance, which was less than 40%, indicating that the common method bias was not present in this study.

### 3.2. Descriptive statistics, inferential statistics, and correlation analyzes

Table 2 shows the means and standard deviations of the main variables and gender differences in smartphone addiction and meaning in life. Specifically, at Time 1, Time 2, and Time 3, female college students’ scores of meaning in life (MIL) were significantly lower than male college students’ scores. Besides, at Time 1, Time 2, and Time 3, female college students’ smartphone addiction (SA) scores were significantly higher than male college students’ scores.

**Table 2:** Gender differences in college students’ smartphone addiction and meaning in life.

	T	*M* ± *SD*		*t*
		Male (*n* = 338)	Female (*n* = 367)	
SA	T1	42.61 ± 5.968	46.21 ± 6.002	−2.558*
	T2	45.09 ± 6.374	48.31 ± 6.521	−2.146*
	T3	42.53 ± 4.561	46.15 ± 6.141	−2.643**
MIL	T1	26.66 ± 1.903	25.27 ± 2.108	2.882**
	T2	25.05 ± 2.726	23.14 ± 2.892	2.852**
	T3	26.36 ± 1.866	25.31 ± 2.078	2.199*

MIL, Meaning in life; SA, Smartphone addiction.

* *p* < 0.05; ** *p* < 0.01.

As shown in Table 3, there was a significant correlation between MIL at time 1, time 2, and time 3 (*r* = 0.48 ~ 0.62) among all participants. There was a significant correlation between SA at time 1, time 2, and time 3 (*r* = 0.69 ~ 0.78) among all participants. At time 1, time 2 and time 3, MIL was significantly correlated with SA (*r* = −0.50 ~ −0.68) among all participants. In addition, the time-dependent correlation between MIL and SA among all participants was also significant (*r* = −0.39 ~ −0.61).

**Table 3:** Inter-correlations of the variables among all participants (*n* = 705).

Variables	1	2	3	4	5	6
1 T1MIL	1					
2 T2MIL	0.48**	1				
3 T3MIL	0.56**	0.62**	1			
4 T1SA	−0.68**	−0.61**	−0.52**	1		
5 T2SA	−0.43**	−0.67**	−0.57**	0.72**	1	
6 T3SA	−0.67**	−0.39**	−0.50**	0.78**	0.69**	1

MIL, Meaning in life; SA, Smartphone addiction; ** *p* < 0.01.

As shown in Table 4, among male college students, there was a significant correlation between MIL at time 1, time 2, and time 3 (*r* = 0.47 ~ 0.60). There was a significant correlation between SA at time 1, time 2, and time 3 (*r* = 0.68 ~ 0.77) among male college students. At time 1, time 2 and time 3, among male college students, MIL was significantly correlated with SA (*r* = −0.46 ~ −0.63). In addition, the time-dependent correlation between MIL and SA among male college students was also significant (*r* = −0.32 ~ −0.47).

**Table 4:** Inter-correlations of the variables among male college students (*n* = 338).

Variables	1	2	3	4	5	6
1 T1MIL	1					
2 T2MIL	0.47**	1				
3 T3MIL	0.55**	0.60**	1			
4 T1SA	−0.57**	−0.41**	−0.49**	1		
5 T2SA	−0.36**	−0.63**	−0.47**	0.70**	1	
6 T3SA	−0.49**	−0.32**	−0.46**	0.77**	0.68**	1

MIL, Meaning in life; SA, Smartphone addiction; ** *p* < 0.01.

As shown in Table 5, there was a significant correlation between MIL at time 1, time 2, and time 3 (*r* = 0.50 ~ 0.66) among female college students. There was a significant correlation between SA at time 1, time 2, and time 3 (*r* = 0.70 ~ 0.80) among female college students. At time 1, time 2 and time 3, among female college students, MIL was significantly correlated with SA (*r* = −0.51 ~ −0.70). Furthermore, among female college students, the time-dependent correlation between MIL and SA was also significant (*r* = −0.52 ~ −0.68).

**Table 5:** Inter-correlations of the variables among female college students (*n* = 367).

Variables	1	2	3	4	5	6
1 T1MIL	1					
2 T2MIL	0.50**	1				
3 T3MIL	0.53**	0.66**	1			
4 T1SA	−0.70**	−0.68**	−0.55**	1		
5T2SA	−0.60**	−0.70**	−0.68**	0.75**	1	
6T3SA	−0.69**	−0.52**	−0.51**	0.80**	0.70**	1

MIL, Meaning in life; SA, Smartphone addiction; ** *p* < 0.01.

### 3.3. Testing the cross-lagged panel model

**Figure 2:**
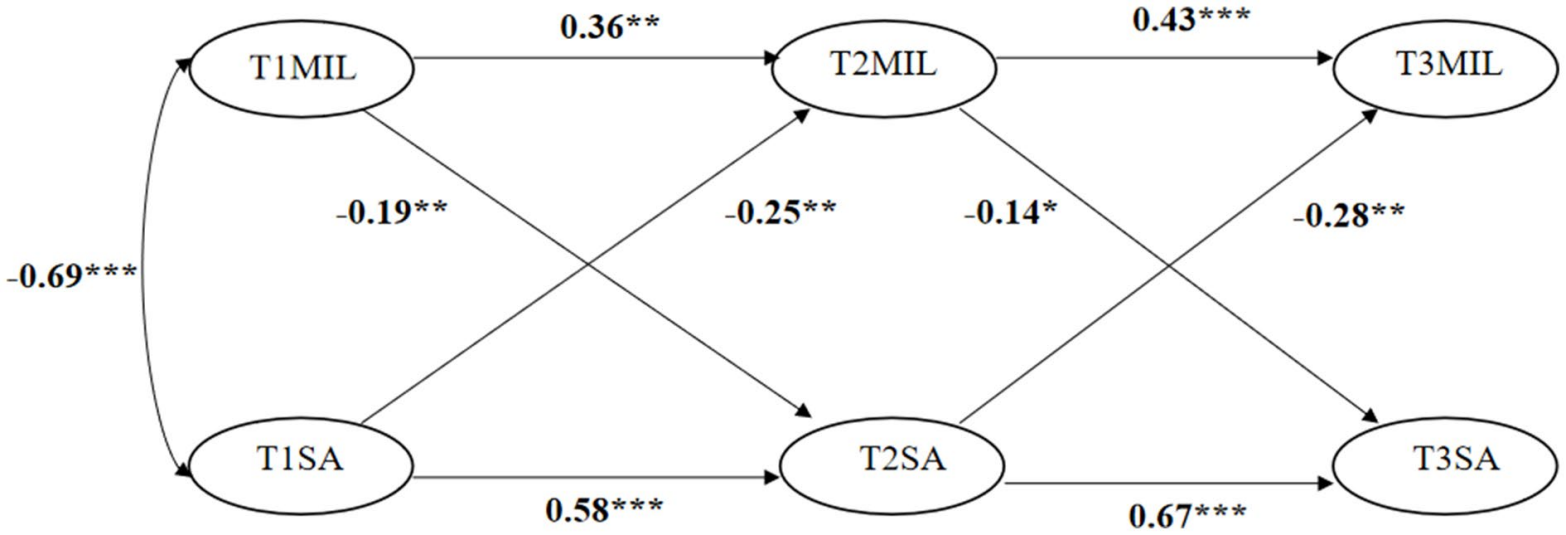
Path diagram of the cross-lagged panel model.

Table 6 shows the results of model comparisons of the four competing models. All models fitted well. However, Model 2 (smartphone addiction effects), Model 3 (meaning in life effects), and Model 4 (reciprocal effects) better fitted the data than did Model 1 (no cross-lagged effects). Model 4 (reciprocal effects) yielded a better model fit than did Model 2 (smartphone addiction effects) and Model 3 (meaning in life effects). Thus, the reciprocal effects model (Model 4) showed the best model fit. The results of cross-lagged panel analysis (see Figure 2) showed that: (a) Meaning in life (MIL) at time 1 could significantly negatively predict smartphone addiction (SA) at time 2 (*β* = −0.19, *p* < 0.01), (b) MIL at time 2 could significantly negatively predict SA at time 3 (*β* = −0.14, *p* < 0.05), (c) SA at time 1 could significantly negatively predict MIL at time 2 (*β* = −0.25, *p* < 0.01), and (d) SA at time 2 could significantly negatively predict MIL at time 3 (*β* = −0.28, *p* < 0.01). These results verified that there were negative reciprocal relationships between MIL and SA.

**Table 6:** Model fit indexes and comparison for different models of the relationship between meaning in life and smartphone addiction among college students.

Model tested	Model fit measures						Model differences			
	*χ^2^*	*df*	CFI	SRMR	TLI	RMSEA	Model comparison	△CFI	△*χ*^2^	△*df*
Model 1: No cross-lagged effects	3375.358	587	0.910	0.047	0.905	0.076				
Model 2: Smartphone addiction effects	3354.232	585	0.933	0.031	0.933	0.055	M2 vs. M1	>0.01	21.126***	2
Model 3: Meaning in life effects	3358.079	585	0.936	0.033	0.934	0.055	M3 vs. M1	>0.01	17.279***	2
Model 4: Reciprocal effects	3342.486	583	0.959	0.029	0.961	0.045	M4 vs. M1	>0.01	32.872***	4
							M4 vs. M2	>0.01	11.746**	2
							M4 vs. M3	>0.01	15.593***	2

Model 1: No cross-lagged effects (T1MIL → T2SA, T2MIL → T3SA, T1SA → T2MIL and T2SA → T3MIL are dropped); Model 2: Smartphone addiction effects (T1SA → T2MIL and T2SA → T3MIL are dropped); Model 3: Meaning in life effects (T1MIL → T2SA and T2MIL → T3SA are dropped); Model 4: Reciprocal effects (all paths are included).

** *p* < 0.01; *** *p* < 0.001.

### 3.4. Multi-group tests by gender on the reciprocal effects model

The measurement model (see Table 7) showed an acceptable fit for male college students and female college students. Thus, the measurement invariance tests can be performed next. As shown in Table 4, Model 1, model 2, and model 3 all fitted well. The model comparison showed that the fit indices of model 2 was not significantly different from that of model 1 (△CFI < 0.01, *p* > 0.05). Further comparing model 3 with model 2, the result found that the fit indices of model 3 was not significantly different from that of model 2 (△CFI < 0.01, p > 0.05). These results indicated that the measurement invariance across gender was established. Thus, structural model comparison can be conducted next.

**Table 7:** Measurement invariance tests for male college students and female college students.

Model tested	Model fit measures						Model differences			
	*χ^2^*	*df*	CFI	SRMR	TLI	RMSEA	△CFI	△*χ*^2^	△*df*	*p*
Separate groups										
Male	1268.551	579	0.968	0.030	0.963	0.041				
Female	1917.278	579	0.953	0.033	0.955	0.049				
Model 1: Configural invariance	3185.829	1,158	0.957	0.036	0.955	0.065				
Model 2: Metric invariance	3215.990	1,188	0.953	0.040	0.952	0.069	<0.01	30.161	30	0.46
Model 3: Scalar invariance	3249.391	1,218	0.950	0.043	0.947	0.071	<0.01	33.401	30	0.31

As shown in Table 8, two cross-lagged paths in the same direction were constrained to be equal between male and female in each comparison model. The results showed that the difference between model 4b and model 4a was significant (△CFI > 0.01, *p* < 0.01). Compared with model 4a, model 4c showed a significant difference (△CFI > 0.01, *p* < 0.01). The above results revealed that the reciprocal effects among female college students were significantly different from that of male college students.

**Table 8:** Multi-group (male vs. female) tests on the reciprocal effects model.

Model tested	Model fit measures						Model differences			
	*χ^2^*	*df*	CFI	SRMR	TLI	RMSEA	Model comparison	△CFI	△*χ*^2^	△*df*
Model 4a: Unconstrained model	3306.619	583	0.953	0.028	0.957	0.035				
Model 4b: Constrain T1MIL → T2SA and T2MIL → T3SA	3316.808	585	0.922	0.048	0.925	0.073	M4b vs. M4a	>0.01	10.189**	2
Model 4c: Constrain T1SA → T2MIL and T2SA → T3MIL	3320.329	585	0.913	0.047	0.920	0.078	M4c vs. M4a	>0.01	13.710**	2

MIL, Meaning in life; SA, Smartphone addiction.

** *p* < 0.01.

### 3.5. Gender differences in reciprocal effects

Comparing the differences of reciprocal effects among male college students (group 1) and female college students (group 2), the results (see Table 9) showed that: (a) The path coefficient of T1MIL → T2SA in female college students were significantly larger than that of male college students (male: *β* = −0.11; female: *β* = −0.32), (b) The path coefficient of T1SA → T2MIL in female college students were significantly larger than that of male college students (male: *β* = −0.17; female: *β* = −0.43), (c) The path coefficient of T2MIL → T3SA in female college students were significantly larger than that of male college students (male: *β* = −0.09; female: *β* = −0.23), and (d) The path coefficient of T2SA → T3MIL in female college students were significantly larger than that of male college students (male: *β* = −0.20; female: *β* = −0.40). In a word, the reciprocal effects among female college students were significantly stronger than that of male college students.

**Table 9:** The results of gender comparison in reciprocal effects.

	Pathway 1: T1MIL → T2SA			Pathway 2: T1SA → T2MIL			Pathway 3: T2MIL → T3SA			Pathway 4: T2SA → T3MIL		
	*β*	SE	*Z*	*β*	SE	*Z*	*β*	SE	*Z*	*β*	SE	*Z*
Group 1: Male	−0.11	0.05	−2.05*	−0.17	0.08	−2.13*	−0.09	0.05	−1.98*	−0.20	0.07	−2.70**
Group 2: Female	−0.32	0.08	−4.09***	−0.43	0.10	−4.47***	−0.23	0.08	−2.78**	−0.40	0.09	−4.26***
Group 1 *VS* Group 2	0.21	0.07	2.86**	0.26	0.09	3.05**	0.14	0.06	2.28*	0.20	0.07	2.91**

MIL, Meaning in life; SA, Smartphone addiction.

* *p* < 0.05; ** *p* < 0.01; *** *p* < 0.001.

## 4. Discussion

A three – wave cross – lagged panel design was used to explore the reciprocal relationships between meaning in life (MIL) and smartphone addiction (SA) among Chinese college students in this study. The findings contribute to a deeper understanding of the dynamic relationships between MIL and SA and the gender differences in these relationships.

### 4.1. Gender differences in college students’ MIL and SA

Consistent with previous cross-sectional studies (49–52), the research results found that the MIL of male college students was significantly stronger than that of female college students at both time 1, time 2, and time 3. The gender differences may be related to the different expectations of males and females in society. In the general concept of Chinese society, males are expected to be more responsible and motivated than females. Under the influence of this social expectation, the setbacks encountered in life may inspire males’ fighting spirit more, from which they will experience a stronger sense of responsibility and experience more MIL in the process of constantly breaking through themselves. On the other hand, compared with males, females tend to be more sensitive and emotional, and they are more likely to take a negative coping style when facing difficulties, resulting in makes it difficult for them to solve them when they encounter difficulties, which is not conducive to obtaining their MIL.

This study also found that there were significant gender differences in SA at time 1, time 2 and time 3, that is, female college students’ smartphone addiction was more serious than that of male college students, which was consistent with many previous studies (53–59). The reason for the above gender differences is probably that males are more independent, while females are more socially dependent (86, 87). Compared with males, in order to satisfy social needs, females are more relational-oriented, and they participate in social activities more frequently. Correspondingly, either in the real world or the virtual world, females were more socially involved than males (87, 88). Also, from the perspective of traditional social culture in China, society is more inclined to expect females to have introverted and implicit personality (89). Under the requirements of this traditional social culture, female college students are more willing to express themselves in an implicit and indirect way, so using mobile social media (e.g., QQ and WeChat) inevitably becomes their best choice. They tend to use smartphones as an important medium of interpersonal communication, and they will communicate and interact with others through QQ, WeChat, and other apps on smartphones. Thus, the probability of female college students’ SA greatly increases.

### 4.2. The reciprocal relationships between MIL and SA

By conducting cross-lagged panel analysis, this study showed that there were negative reciprocal relationships between MIL and SA. Specifically, MIL at time 1 could significantly negatively predict SA at time 2, and MIL at time 2 could significantly negatively predict SA at time 3. These findings demonstrated that MIL is an important influencing factor of college students’ SA, which is congruent with meaning therapy theory (30). Frankl (30) proposed that finding the meaning of life is the primary motivation of human beings. If individuals cannot find the value and meaning of their existence, they will develop a sense of empty existence and easily lose themselves in social life, which may lead to addictive behaviors (30). Besides, the above findings are also supported by some previous studies among college students. Some scholars found that MIL could positively affect the addictive behavior of college students (28, 29). A recent intervention study on SA based on meaning therapy theory verified that meaningful therapy was an effective method to improve SA (90), which further demonstrated that MIL is an important predictor of SA. The higher the level of college students’ MIL, the stronger their school adjustment may be, and the lower the possibility of their SA. Contrariwise, if college students lack MIL, they are likely to have school adjustment problems and show depression and negativity. In order to relieve negative emotions and make up for inner emptiness, college students are easily addicted to the “virtual network world” of smartphones (32).

Furthermore, this study found that SA at time 1 could significantly negatively predict MIL at time 2, and SA at time 2 could significantly negatively predict MIL at time 3. These findings demonstrated that SA also influenced college students’ MIL. According to resource conservation theory, individuals have limited internal and external resources, and when these resources are consumed without return, they are likely to affect people’s normal lives and lead to negative consequences (33). If college students consume a lot of resources using their smartphones, they will have very limited or even few resources to cope with real life. Specifically, addiction to smartphones occupies a large number of individual cognitive resources, when there are multiple cognitive tasks (e.g., talking while playing smartphones; learning while playing smartphones) at the same time, individuals are prone to cognitive overload, which easily leads to their cognitive failure (91, 92). If this situation continues, it will inevitably have a negative impact on the physical and mental health of individuals. Thus, it can be well understood that college students will have interpersonal problems and negative emotions due to excessive use of smartphones or even addiction to smartphones, which will eventually lead to the decline of college students’ MIL.

### 4.3. The moderating effect of gender

By comparing the differences of reciprocal effects among male college students and female college students, this study found that the reciprocal effects in female college students were significantly stronger than those of male college students. Specifically, the influence of MIL on SA among female college students was significantly stronger than that of male college students. The gender differences in the above relationships can be explained from the perspective of gender differences in coping with stress. When dealing with external stressors, males may be more inclined to cope with stress using a positive coping style that focuses on problem solving. Yet females often feel they are in stressful situations and have more chronic stress than males, and they may be more inclined to cope with stress using negative coping styles that are not conducive to problem solving (64, 65). It should be emphasized that negative coping styles are not conducive to problem solving and may further exacerbate the negative effects of stress on female college students (65), thus leading to a lower level of MIL for female students than for male students. Since smartphones have the aforementioned functional characteristics, it is easier to get alternative satisfaction and compensation by using them (32). Thus, in order to make up for the psychological emptiness caused by the low level of MIL and alleviate negative emotions, female college students may use smartphones more frequently than male college students, which leads to more serious SA among female college students.

Meanwhile, this study have also shown that SA had a significantly stronger effect on female college students’ MIL than male college students. According to the social replacement hypothesis, the excessive use of mobile social media may replace activities in real situations, thus occupying individuals’ time and energy for offline activities, especially reducing the opportunities for offline communication with others, which will destroy individuals’ offline social support system, reduce the channels for individuals to obtain other people’s information, and make them participate in offline social activities insufficiently (72). In the end, it may lead to the reduction or lack of individual beneficial experiences, thus leading to negative emotions (93). Previous studies have found that males mainly use smartphones for more utilitarian and/or entertainment reasons, while females frequently use smartphones to meet social needs (55). Compared with males, females are more inclined to seek social support and are more sensitive (94). Therefore, SA may damage the social support system of female college students in real life and have a negative impact on their social interaction in real life, resulting in a lower level of MIL for female college students than for male college students. In a sense, the psychological need satisfaction that smartphone-addicted female college students seek in using their smartphones is a false substitute satisfaction that often serves to protect themselves from the aggression that comes with interpersonal and emotional problems. Although they find themselves in the virtual world for a short period of time, they experience more negative emotions in real life, which does not provide motivation for their development but damages their physical and mental health and makes them lose their MIL.

## 5. Conclusion

In this study, the three-wave cross-lag panel design was used to explore the reciprocal relationships between meaning in life (MIL) and college students’ smartphone addiction (SA). The results showed that MIL could negatively affect college students’ SA, and SA could also negatively affect college students’ MIL. In addition, there are significant gender differences in the reciprocal relationships between MIL and college students’ SA. Female college students are more likely to be addicted to smartphones because of their low level of MIL than that of male college students, which in turn leads to their further decline in MIL. This study not only deepens the research on the relationship between MIL and SA, but also provides theoretical support for preventing and intervening with college students’ smartphone addiction from a gender difference perspective.

## 6. Limitations, future directions, and implications

There are inevitably some limitations despite the fact that this study has produced some valuable findings. First, the participants in this study are all college students in China, and it is important to examine whether the findings consistent with this study exist in other countries and cultures in the future. Secondly, the participants in this study are mainly college students in northern China, and the current findings may not be applicable to all college students in China. Future research needs to expand the sampling range to examine whether the findings are applicable to college students in other parts of China. Thirdly, this study only explores the reciprocal relationships between meaning in life (MIL) and college students’ smartphone addiction (SA) by using the cross-lag panel design, and future research needs to use the cross-lag panel design to explore the complex mechanism of the influence of MIL on SA and SA on MIL.

The findings of this study have positive significance. As for the theoretical significance, this study examines the reciprocal relationships between MIL and college students’ SA and the moderating effect of gender on the reciprocal relationships for the first time, which can further deepen the research on the relationship between MIL and college students’ SA. Regarding the practical significance, this study can provide guidance for the targeted prevention and intervention with college students’ SA and lack of MIL. Specifically, we can prevent and intervene with college students’ SA by improving their MIL, especially that of female college students. In addition, the negative influence of SA on college students’ MIL can be reduced by interfering with their SA, especially that of female college students.

## Data availability statement

The raw data supporting the conclusions of this article will be made available by the authors, without undue reservation.

## Ethics statement

The studies involving human participants were reviewed and approved by Research Ethics Committee of Shandong Women's University. The patients/participants provided their written informed consent to participate in this study.

## Author contributions

HZ and TS: methodology, investigation, formal analysis, writing—original draft, editing, and translation. SR-G: revised, validation, and supervision. MF: writing—review and supervision. All authors contributed to the article and approved the submitted version.

## Conflict of interest

The authors declare that the research was conducted in the absence of any commercial or financial relationships that could be construed as a potential conflict of interest.

## Publisher’s note

All claims expressed in this article are solely those of the authors and do not necessarily represent those of their affiliated organizations, or those of the publisher, the editors and the reviewers. Any product that may be evaluated in this article, or claim that may be made by its manufacturer, is not guaranteed or endorsed by the publisher.

## References

[1] China Internet Network Information Center. China statistical report on internet development (no. 50). (2022). Available at: http://www.cnnic.net.cn/n4/2022/0914/c88-10226.html (accessed September 2, 2022).

[2] Ahn J Jung Y. The common sense of dependence on smartphone: a comparison between digital natives and digital immigrants. New Media Soc. (2016) 18:1236–56. doi: 10.1177/1461444814554902

[3] Sunday OJ Adesope OO Maarhuis PL. The effects of smartphone addiction on learning: a meta-analysis. Comp Hum Behav Rep. (2021) 4:100114. doi: 10.1016/j.chbr.2021.100114

[4] Hughes N Burke J. Sleeping with the frenemy: how restricting bedroom use of smartphones impacts happiness and well being. Comput Hum Behav. (2018) 85:236–44. doi: 10.1016/j.chb.2018.03.047

[5] Li MJ. Influence of mobile phone use on college students’ physical and mental health. Unpublished master’s thesis. Changchun: Jilin University (2016).

[6] Haug S Castro RP Kwon M Filler A Kowatsch T Schaub MP. Smartphone use and smartphone addiction among young people in Switzerland. J Behav Addict. (2015) 4:299–307. doi: 10.1556/2006.4.2015.037 26690625 PMC4712764

[7] Lopez-Fernandez O. Short version of the smartphone addiction scale adapted to Spanish and French: towards a cross-cultural research in problematic mobile phone use. Addict Behav. (2017) 64:275–80. doi: 10.1016/j.addbeh.2015.11.013, PMID: 26685805

[8] Aljomaa SS Qudah MFA Albursan IS Bakhiet SF Abduljabbar AS. Computers in human behavior smartphone addiction among university students in the light of some variables. Comput Hum Behav. (2016) 61:155–64. doi: 10.1016/j.chb.2016.03.041

[9] Kim HJ Min JY Kim HJ Min KB. Accident risk associated with smartphone addiction: a study on university students in Korea. J Behav Addict. (2017) 6:699–707. doi: 10.1556/2006.6.2017.070, PMID: 29099234 PMC6034962

[10] Mohammadi S Valinejadi A Saman JA Karimpour H Kaivanfar M Safaeipour M. Assessment of addiction to internet, smartphone and social networks among students of medical sciences: a cross sectional study. Electron J Gen Med. (2018) 15:em35. doi: 10.29333/ejgm/85685

[11] Tateno M Kim DJ Teo AR Skokauskas N Guerrero APS Kato TA. Smartphone addiction in Japanese college students: usefulness of the japanese version of the smartphone addiction scale as a screening tool for a new form of internet addiction. Psychiatry Investig. (2019) 16:115–20. doi: 10.30773/pi.2018.12.25.2, PMID: 30808117 PMC6393743

[12] Ching SM Lee KW Yee A Sivaratnam D Suppiah S. The malay version of smartphone addiction scale: development, factor structure and validation of a short form for malaysian adolescents. Med J Malays. (2020) 75:561–7. PMID: 32918427

[13] Cao L Lin JL Mo JF. Influence factors of mobile phone addiction among college students in Hainan province: a structural equation model-based analysis. Chin J Public Health. (2021) 37:82–5. doi: 10.11847/zgggws1124991

[14] Dietz S Henrich C. Texting as a distraction to learning in college students. Comput Hum Behav. (2014) 36:163–7. doi: 10.1016/j.chb.2014.03.045

[15] Volkmer SA Lermer E. Unhappy and addicted to your phone? – higher mobile phone use is associated with lower well-being. Comput Hum Behav. (2018) 93:210–8. doi: 10.1016/j.chb.2018.12.015

[16] Kozan HİÖ Kavaklı M Ak M Kesici Ş. The associations among smartphone addiction, general belongingness and happiness: a structural equation modelling. Klin Psikiyatr Derg. (2019) 22:436–44. doi: 10.5505/KPD.2019.87587

[17] Satici B Deniz ME. Modeling emotion regulation and subjective happiness: smartphone addiction as a mediator. Addicta. (2020) 7:146–52. doi: 10.5152/addicta.2020.20035

[18] Zhang YL Lu GZ Song XM Huang HJ Ma XY Zhang YH. Mediating effect of self-control and interpersonal adaptability on relationship between self-esteem and mobile phone addiction tendency in college students. Chin Ment Health J. (2018) 32:420–4. doi: 10.3969/j.issn.1000-6729.2018.05.013

[19] Zhang YL Lv GZ Jin TL Li S Jiang HB Liang L. The effect of college students' smartphone addiction tendency on their interpersonal adaptability: the intermediary role of alexithymia. Chin J Spec Educ. (2018) 66:83–8. doi: 10.3969/j.issn.1007-3728.2018.02.015

[20] Wang X. Study on the correlation between depression symptoms, cell phone dependence and suicidal ideation of students in a vocational school. Chin J Prev Med. (2017) 18:609–12. doi: 10.3389/fpubh.2021.809463

[21] Ismail WSW Sim ST Tan KA Bahar N Ibrahim N Mahadevan R. The relations of internet and smartphone addictions to depression, anxiety, stress, and suicidality among public university students in Klang Valley. Malaysia Perspect Psychiatr Care. (2020) 56:949–55. doi: 10.1111/ppc.12517 32363648

[22] Lee J Ahn JS Min S Kim MH. Psychological characteristics and addiction propensity according to content type of smartphone use. Int J Environ Res Public Health. (2020) 17:2292. doi: 10.3390/ijerph17072292, PMID: 32235319 PMC7177936

[23] Steger MF Frazier P Oishi S Kaler M. The meaning in life questionnaire: assessing the presence of and search for meaning in life. J Couns Psychol. (2006) 53:80–93. doi: 10.1037/0022-0167.53.1.80

[24] Thege BK Urban R Kopp MS. Four-year prospective evaluation of the relationship between meaning in life and smoking status. Abus Treat Prev Pol. (2013) 8:1–5. doi: 10.1186/1747-597X-8-8 PMC3598484 23433067

[25] Eryilmaz A. Meaning of life-setting life goals: comparison of substance abusers and non-abusers. Turk Psychol Couns Guid J. (2014) 5:235–43.

[26] Zhang Y Mei SL Li L Chai JX Li JM Du HY. The relationship between impulsivity and internet addiction in Chinese college students: a moderated mediation analysis of meaning in life and self-esteem. PLoS One. (2015) 10:e0131597. doi: 10.1371/journal.pone.0131597, PMID: 26171860 PMC4501566

[27] Yao MP Jia ZB Chen X Jiao SS. The mediating effect of meaning in life on boredom and mobile phone dependence behavior of college students. Chin J Sch Health. (2016) 37:453–6. doi: 10.16835/j.cnki.1000-9817.2016.03.040

[28] Çevik C Ciğerci Y Kılıç I Uyar S. Relationship between smartphone addiction and meaning and purpose of life in students of health sciences. Perspect Psychiatr Care. (2020) 56:705–11. doi: 10.1111/ppc.12485, PMID: 32065417

[29] Hu Q Liu QX Wang ZY. Meaning in life as a mediator between interpersonal alienation and smartphone addiction in the context of Covid-19: a threewave longitudinal study. Comput Hum Behav. (2022) 127:107058. doi: 10.1016/j.chb.2021.107058, PMID: 34690416 PMC8519895

[30] Frankl VE. Man’s Search for Meaning: An Introduction to Logotherapy. Boston: Beacon Press (1963).

[31] Zhang XG Qin J Huang WY. Self-control mediates the relationship between the meaning in life and the mobile phone addiction tendency of Chinese college students. Chin J Stud Psychol Behav. (2019) 17:536–45. doi: 10.3969/j.issn.1672-0628.2019.04.013

[32] Zhao H Rafik-Galea S Fitriana M Song T. Meaning in life and smartphone addiction among Chinese female college students: the mediating role of school adjustment and the moderating role of grade. Front Psychol. (2023) 14:1092893. doi: 10.3389/fpsyg.2023.1092893, PMID: 36818081 PMC9928732

[33] Hobfoll SE. Conservation of resources. A new attempt at conceptualizing stress. Am Psychol. (1989) 44:513–24. doi: 10.1037/0003-066X.44.3.513, PMID: 2648906

[34] Chen L Yan Z Tang WJ Yang FY Xie XD He JC. Mobile phone addition levels and negative emotions among Chinese young adults: the mediating role of interpersonal problems. Comput Hum Behav. (2016) 55:856–66. doi: 10.1016/j.chb.2015.10.030

[35] Bi TY Kou H Xie QH Dong J. Mediating roles of social anxiety and interpersonal distress in the relationship between mobile phone addiction and loneliness. J Psychol Afr. (2022) 32:487–93. doi: 10.1080/14330237.2022.2121058

[36] Mohamed SM Mostafa MH. Impact of smartphone addiction on depression and self-esteem among nursing students. Nurs Open. (2020) 7:1346–53. doi: 10.1002/nop2.506, PMID: 32802355 PMC7424452

[37] Ding YM Wan X Lu GL Huang HT Liang YP Yu JF. The associations between smartphone addiction and self-esteem, self-control, and social support among Chinese adolescents: a meta-analysis. Front Psychol. (2022) 13:1029323. doi: 10.3389/fpsyg.2022.1029323, PMID: 36420390 PMC9677120

[38] Li Y Li GX Liu L Wu H. Correlations between mobile phone addiction and anxiety, depression, impulsivity, and poor sleep quality among college students: a systematic review and meta-analysis. J Behav Addict. (2020) 9:551–71. doi: 10.1556/2006.2020.00057, PMID: 32903205 PMC8943681

[39] Diao YC Feng ZY Ma HF Liu MH Zhao S Long MJ. Loneliness and anxiety among Chinese medical students: the mediating role of mobile phone addiction and the moderating role of gender. Soc Sci Comput Rev. (2022) 41:482–94. doi: 10.1177/08944393221106934

[40] Lv JP Ren H Qin ZY Hu YY Cao RL Liang LL. Alexithymia and mobile phone addiction among college students with and without siblings: a moderated mediation of depression and gender. Int J Mental Health Addict. (2022). doi: 10.1007/s11469-022-00761-w

[41] Feng ZY Diao YC Ma HF Liu MH Long MJ Zhao S. Mobile phone addiction and depression among Chinese medical students: the mediating role of sleep quality and the moderating role of peer relationships. BMC Psychiatry. (2022) 22:567. doi: 10.1186/s12888-022-04183-9, PMID: 35999533 PMC9396829

[42] Zhang KX Guo HY Wang TL Zhang JH Yuan GJ Ren J. A bidirectional association between smartphone addiction and depression among college students: a cross-lagged panel model. Front Public Health. (2023) 11:1083856. doi: 10.3389/fpubh.2023.1083856, PMID: 36761134 PMC9902510

[43] Jun S. The reciprocal longitudinal relationships between mobile phone addiction and depressive symptoms among Korean adolescents. Comput Hum Behav. (2016) 58:179–86. doi: 10.1016/j.chb.2015.12.061

[44] Lin CC. Gratitude and suicidal ideation in undergraduates in Taiwan: the mediating role of self-esteem and meaning in life. Omega-J Death Dying. (2019) 84:177–93. doi: 10.1177/0030222819882845 31623525

[45] Sab LH Ghomian S. Prediction of meaning of life in student based on spirituality, self-esteem, and positive affect. Revista Inclusiones. (2019) 6:535–42.

[46] Ye JH Ye XT. Adolescents' interpersonal relationships, self-consistency, and congruence: life meaning as a mediator. J Soc Behav Pers. (2020) 48:e9428:1–11. doi: 10.2224/sbp.9428

[47] Li YD Peng YH Wang ZY Deng YS Zhou WY Liu Z. Influence of anxiety and depression on college students' meaning in life: the mediating role of boredom tendency. Chin J Behav Med Brain Sci. (2021) 30:634–9. doi: 10.3760/cma.j.cn371468-20210319-00148

[48] Chen M Huang SH Xu N Li JJ Wu QL. Effect between trait anxiety and excessive WeChat use in college students: the chain mediating role of cognitive reappraisal and meaning in life. Chin J Health Psychol. (2023) 31:249–54.

[49] Wang JY. Study on Death Attitudes and its Relationship with Self-Esteem and Meaning of Life Among Undergraduate Students. Unpublished master’s thesis. Changchun: Jilin University, Harbin Normal University (2013).

[50] Zhou FJ Fan N Wang YC. Relationship among the big five factors personality, psychological capital and meaning in life of college students. Chin J Health Psychol. (2015) 23:1866–71.

[51] Zhou J. The Current Situation of Chongqing College Students' Life Meaning Feeling Investigation and Intervention Study. Unpublished master’s thesis. Changchun: Jilin University. Chongqing Normal University (2016).

[52] Zhou Y. The Relationship and Intervention of Meaning in Life, Resilience, Perceived Stress and Mobile Phone Dependence. Unpublished master’s thesis. Shanghai: East China Normal University (2022).

[53] Walsh SP White KM Cox S Young RMD. Keeping in constant touch: the predictors of young Australians’ mobile phone involvement. Comput Hum Behav. (2011) 27:333–42. doi: 10.1016/j.chb.2010.08.011

[54] Mok JY Choi SW Kim DJ Choi JS Lee J Ahn H. Latent class analysis on internet and smartphone addiction in college students. Neuropsychiatr Dis Treat. (2014) 10:817–28. doi: 10.2147/NDT.S59293, PMID: 24899806 PMC4038421

[55] Roberts JA Yaya LHP Manolis C. The invisible addiction: cell-phone activities and addiction among male and female college students. J Behav Addict. (2014) 3:254–65. doi: 10.1556/JBA.3.2014.015, PMID: 25595966 PMC4291831

[56] Zhu LL Liu ZQ. Current situation and characteristics of mobile phone addiction of higher vocational college students. Chin J Health Stat. (2017) 34:767–9.

[57] Nayak JK. Relationship among smartphone usage, addiction, academic performance and the moderating role of gender: a study of higher education students in India. Comput Educ. (2018) 123:164–73. doi: 10.1016/j.compedu.2018.05.007

[58] Yi Q Luwen Z Xiao Z Hui W Yuting Z Jinhai S. Cell phone addiction and apps activities among Chinese medical students: prevalence and risk factors. J Men's Health. (2020) 16:27–38. doi: 10.15586/JOMH.V16I2.170

[59] He W Xia Y. A study on the relationship between mobile phone addiction, psychological needs and mobile phone use of college students. Chin J West Norm Univ. (2021) 46:105–11.

[60] Miller SM Kirsch N. Sex differences in cognitive coping with stress In: Barnett RC Biener L Baruch GK, editors. Gender & Stress. New York: The Free Press (1987). 278–307.

[61] Ptacek JT Smith RE Zanas J. Gender, appraisal, and coping: a longitudinal analysis. J Pers. (1992) 60:747–70. doi: 10.1111/j.1467-6494.1992.tb00272.x

[62] Almeida DM Kessler RC. Everyday stressors and gender differences in daily distress. J Pers Soc Psychol. (1998) 75:670–80. doi: 10.1037/0022-3514.75.3.670 9781406

[63] McDonough P Walters W. Gender and health: reassessing patterns and explanations. Soc Sci Med. (2001) 52:547–59. doi: 10.1016/S0277-9536(00)00159-3, PMID: 11206652

[64] Matud MP. Gender differences in stress and coping styles. Pers Individ Differ. (2004) 37:1401–15. doi: 10.1016/j.paid.2004.01.010

[65] Al-Bahrani M Aldhafri S Alkharusi H Kazem A Alzubiadi A. Age and gender differences in coping style across various problems: Omani adolescents' perspective. J Adolesc. (2013) 36:303–9. doi: 10.1016/j.adolescence.2012.11.007, PMID: 23395184

[66] Lai XF Jiang XM. The study on the relationship of life events, coping styles and meaning of life among college students. Chin J Chongqing Univ Technol. (2013) 27:119–23.

[67] Chen KR. The Research on the Relationship Between Life Meaning, Coping Style, and Mobile Phone Dependency Among University Students. Unpublished master’s thesis. Changsha: Hunan Normal University (2017).

[68] Kwon M Lee JY Won WY Park JW Min JA Hahn C. Development and validation of a smartphone addiction scale (SAS). PLoS One. (2013a) 8:e56936. doi: 10.1371/journal.pone.0056936, PMID: 23468893 PMC3584150

[69] Kwon M Kim DJ Cho H Yang S. The smartphone addiction scale: development and validation of a short version for adolescents. PLoS One. (2013b) 8:e83558. doi: 10.1371/journal.pone.0083558, PMID: 24391787 PMC3877074

[70] Lin YH Chang LR Lee YH Tseng HW Kuo TB Chen SH. Development and validation of the smartphone addiction inventory (SPAI). PLoS One. (2014) 9:e98312. doi: 10.1371/journal.pone.0098312, PMID: 24896252 PMC4045675

[71] Wu AMS Cheung VI Ku L Hung EPW. Psychological risk factors of addiction to social networking sites among Chinese smartphone users. J Behav Addict. (2013) 2:160–6. doi: 10.1556/JBA.2.2013.006, PMID: 25215198 PMC4117295

[72] Kraut R Lundmark V Patterson M Kiesler S Mukopadhyay T Scherlis W. Internet paradox: a social technology that reduces social involvement and psychological well-being? Am Psychol. (1998) 53:1017–31. doi: 10.1037/0003-066X.53.9.1017 9841579

[73] Morahan-Martin J Schumacher P. Loneliness and social uses of the internet. Comput Hum Behav. (2003) 19:659–71. doi: 10.1016/S0747-5632(03)00040-2

[74] Williams D Ducheneaut N Xiong L Yee N Nickell E. From tree house to barracks: the social life of guilds in world of Warcraft. Games Cult. (2006) 1:338e361. doi: 10.1177/1555412006292616

[75] Moody EJ. Internet use and its relationship to loneliness. Cyberpsychol Behav. (2001) 4:393–401. doi: 10.1089/109493101300210303 11710265

[76] Cummings J Butler B Kraut R. The quality of online social relationships. Commun ACM. (2002) 45:103–8. doi: 10.1145/514236.514242

[77] Liang LC Zhou D Yuan CY Shao AH Bian YF. Gender differences in the relationship between internet addiction and depression: a cross-lagged study in Chinese adolescents. Comput Hum Behav. (2016) 63:463–70. doi: 10.1016/j.chb.2016.04.043

[78] Jenaro C Flores N Gómez-Vela M González-Gil F Caballo C. Problematic internet and cell-phone use: psychological, behavioral, and health correlates. Addict Res Theory. (2007) 15:309–20. doi: 10.1080/16066350701350247

[79] Van Deursen AJAM Bolle CL Hegner SM Kommers PAM. Modeling habitual and addictive smartphone behavior: the role of smartphone usage types, emotional intelligence, social stress, self-regulation, age, and gender. Comput Hum Behav. (2015) 45:411–20. doi: 10.1016/j.chb.2014.12.039

[80] Liu SS Gan YQ. Reliability and validity of Chinese version of meaning in life scale among college students. Chin Ment Health J. (2010) 24:478–82. doi: 10.3969/j.issn.10006729.2010.06.021

[81] Podsakoff PM MacKenzie SB Lee JY Podsakoff NP. Common method biases in behavioral research: a critical review of the literature and recommended remedies. J Appl Psychol. (2003) 88:879–903. doi: 10.1037/0021-9010.88.5.879, PMID: 14516251

[82] Little TD. Longitudinal Structural Equation Modeling. New York, NY: Guilford Press (2013).

[83] Cheung GW Rensvold RB. Evaluating goodness-of-fit indexes for testing measurement invariance. Struct Equ Model. (2002) 9:233–55. doi: 10.1207/S15328007SEM0902_5

[84] Chen FF. Sensitivity of goodness of fit indexes to lack of measurement invariance. Struct Equ Model. (2007) 14:464–504. doi: 10.1080/10705510701301834

[85] Hair JF Black WC Babin BJ Anderson RE. Multivariate Data Analysis. 7th ed. London: Pearson Education (2014).

[86] Furman W. The measurement of friendship perceptions: conceptual and methodological issues In: Bukowski WM Andrew NF, editors. The company they keep: Friendship in childhood and adolescence. New York, NY: Cambridge University Press (1998). 41–65.

[87] Ang CS. Internet habit strength and online communication: exploring gender differences. Comput Hum Behav. (2017) 66:1–6. doi: 10.1016/j.chb.2016.09.028

[88] Bonetti L Campbell MA Gilmore L. The relationship of loneliness and social anxiety with children's and adolescents' online communication. Cyberpsychol Behav. (2010) 13:279–85. doi: 10.1089/cyber.2009.0215, PMID: 20557247

[89] Zhu LL Liu ZQ. Current situation and characteristics of mobile phone addiction among college students in higher vocational colleges. Chin J Health Statist. (2017) 34:767–9.

[90] Wang JJ. Intervention Study of Group Counseling Based on Meaning Therapy on Teenagers' Smartphone Addiction. Unpublished master’s thesis. Changsha: Hunan Normal University (2017).

[91] Head J Helton WS. Sustained attention failures are primarily due to sustained cognitive load not task monotony. Acta Psychol. (2014) 153:87–94. doi: 10.1016/j.actpsy.2014.09.007 25310454

[92] Zhang B Peng Y Luo XS Mao HL Luo YH Hu RT. Mobile phone addiction and cognitive failures in Chinese adolescents: the role of rumination and mindfulness. J Psychol Afr. (2021) 31:49–55. doi: 10.1080/14330237.2020.1871239

[93] Duvenage M Correia H Uink B Barber BL Donovan CL Modecki KL. Technology can sting when reality bites: Adolescents' frequent online coping is ineffective with momentary stress. Comput Hum Behav. (2020) 102:248–59. doi: 10.1016/j.chb.2019.08.024

[94] Nolen-Hoeksema S. Emotion regulation and psychopathology: the role of gender. Annu Rev Clin Psychol. (2012) 8:161–87. doi: 10.1146/annurev-clinpsy-032511-143109 22035243

